# Examining the Social Porosity of Environmental Features on Neighborhood Sociability and Attachment

**DOI:** 10.1371/journal.pone.0084544

**Published:** 2014-01-10

**Authors:** John R. Hipp, Jonathan Corcoran, Rebecca Wickes, Tiebei Li

**Affiliations:** 1 University of California Irvine, Irvine, California, United States of America; 2 University of Queensland, Brisbane, Queensland, Australia; 3 Griffith University, Brisbane, Queensland, Australia; Institut Pluridisciplinaire Hubert Curien, Franc

## Abstract

The local neighborhood forms an integral part of our lives. It provides the context through which social networks are nurtured and the foundation from which a sense of attachment and cohesion with fellow residents can be established. Whereas much of the previous research has examined the role of social and demographic characteristic in relation to the level of neighboring and cohesion, this paper explores whether particular environmental features in the neighborhood affect social porosity. We define social porosity as the degree to which social ties flow over the surface of a neighborhood. The focus of our paper is to examine the extent to which a neighborhood's environmental features impede the level of social porosity present among residents. To do this, we integrate data from the census, topographic databases and a 2010 survey of 4,351 residents from 146 neighborhoods in Australia. The study introduces the concepts of *wedges* and *social holes*. The presence of two sources of *wedges* is measured: rivers and highways. The presence of two sources of *social holes* is measured: parks and industrial areas. Borrowing from the geography literature, several measures are constructed to capture how these features collectively carve up the physical environment of neighborhoods. We then consider how this influences residents' neighboring behavior, their level of attachment to the neighborhood and their sense of neighborhood cohesion. We find that the distance of a neighborhood to one form of *social hole*–industrial areas–has a particularly strong negative effect on all three dependent variables. The presence of the other form of *social hole*–parks–has a weaker negative effect. Neighborhood *wedges* also impact social interaction. Both the length of a river and the number of highway fragments in a neighborhood has a consistent negative effect on neighboring, attachment and cohesion.

## Introduction

Individuals spend a large part of their lives within their local neighborhood. Therefore the neighborhood context is important because it provides a space to develop social networks and can generate a sense of attachment and cohesion among residents. Consequently, many studies have attempted to capture the features of the neighborhood that promote socializing and social cohesion; for examples, see [1]–[3]. This literature has typically assessed which social and demographic features are characteristic of neighborhoods with higher levels of neighboring and cohesion. By “neighboring”, these studies refer to the idea of residents visiting with each other, reciprocating favors, providing advice, or engaging in various social activities together. Although these studies have provided considerable insights, they have generally tended to overlook the possible importance of physical characteristics in the environment for impacting these social processes. And whereas there is a growing literature focusing on the effect of physical features at relatively micro-scales (for example, the focal or adjacent blocks) for tie formation and social cohesion, studies typically do not focus on the effect of the physical environment at more meso-scales (i.e. the broader neighborhood, or even adjacent neighborhoods).

Given the evidence that social ties are formed based on a distance decay function [4]–[7], it is plausible that the presence of certain physical characteristics nearby can impact neighborhood sociability and cohesion. In this study, we adopt the approach of considering neighborhoods as ecological units of analysis and examine how the physical characteristics of these neighborhoods might impact upon neighborhood sociability and attachment. We specifically focus on how two types of characteristics–what we term *social holes* and *wedges*–can carve up these neighborhoods, and the subsequent effect this has on social behavior. We consider two dimensions of social holes: the presence of industrial areas and parks. We also consider how two types of wedges might impact neighborhoods: rivers and highways. The geographic location and distribution within a neighborhood of these social holes and wedges are likely to impose varying consequences for the social life of a neighborhood. Borrowing from the geography literature, we consider several possible measures that might capture how these social holes and wedges physically impact neighborhoods, and then assess their relative effects on neighborhood perceptions and social behaviors.

In what follows, we first consider the general literature on neighborhood sociability and attachment. We then briefly review the more recent New Urbanism literature which suggests that certain physical characteristics might have important effects on neighboring and cohesion among residents. We next describe our study site and data, and how we construct our measures of interest. This is followed by a presentation of the results from the multilevel models, and closes with a discussion and a consideration of their implications in terms of neighborhood sociability and attachment.

## Background

In the early part of the twentieth century, many scholars suggested that industrialization and increases in mobility and immigration had serious implications for the development of social networks and shared collective norms [8]–[10]. Most prominently, Louis Wirth [10] argued that the large population size, population density, and heterogeneity of cities would result in residents feeling more dislocated and unattached. Yet studies that followed Wirth's seminal work did not find that social relations were reduced due to the urbanization of the population [11]–[13]. Instead, research demonstrated that geographic subareas within metropolitan areas (i.e. neighborhoods) appeared to foster a sense of cohesion and close ties among residents. Sometimes dubbed the “urban village” model, this perspective argued that residents created numerous ties with others living near them (in the same “neighborhood”), and felt a sense of attachment to, and cohesion with, this neighborhood, despite the fact that they resided in a larger metropolitan area. Thus, although the metropolitan area was largely composed of “strangers” to any given resident, this was not consequential given that a resident could form close ties with those living within the same small geographic area. These early findings gave rise to a large body of literature that attempted to identify the social characteristics of neighborhoods that were most important for social cohesion and the density of neighborhood social ties [1], [2], [14]–[17].

This literature has produced numerous insights, For example, studies show that higher levels of racial/ethnic heterogeneity in the neighborhood can negatively impact the degree of socializing [15], [18] and the general sense of cohesion in the neighborhood [14]. Other research suggests that economic inequality is a source of difference among residents that can inhibit socializing and attachment to the neighborhood [17]. Following the systemic model [16] research also indicates that increasing time spent in a neighborhood can bring about a greater sense of familiarity among residents [17], higher levels of neighboring [1], [2], [14], [19]–[21] and facilitate greater cohesion among residents in the neighborhood [1], [2], [22].

Although much sociological scholarship identifies the social-demographic characteristics of neighborhoods that lead to higher cohesion and more social ties, less is known about the physical characteristics of these neighborhoods and their impact on neighborly sociability. We argue there are theoretical reasons to suspect that the physical characteristics of a community can affect social interactions and cohesion. For example, the growing New Urbanism literature considers design features that potentially act as facilitators and barriers to socializing, including the presence of sidewalks and aesthetic features such as trees. Studies indicate that higher levels of housing density, along with shorter blocks, increases the amount of walking by residents [23], [24], which may then encourage contact with fellow residents. Likewise, the physical feature of front porches on units is hypothesized to enhance interaction with neighborhood residents [25]. Further, studies show that greater use of local facilities in neighborhoods (e.g., shopping, recreation, and worship) increases resident interaction [26]. Lund's [27] research provides evidence of this by demonstrating the strong relationship between the presence of local amenities, such as retail outlets and parks, and increased pedestrian travel and resident interaction.

Although there is a growing literature asking whether the physical environment can impact neighboring and cohesion, this research typically focuses on the more micro-physical environment. For example, one study asked whether perceptions of the local environment impacted social participation of older results in a convenience sample in Montreal [28]. Another study, using a non-random sample, viewed correlations between the built form reported social capital (e.g., trusting neighbors, volunteering, attending clubs, etc) [29]. An exploratory study of Perth viewed the relationship between the street network design or nearby amenities and reported social capital [30].

While the New Urbanism literature provides some evidence to support the link between the physical features of place and the sociability of residents, this body of work focuses more on micro features of the environment, and therefore gives less consideration to possible meso or macro level physical features that might impact neighborhood cohesion and networks. Furthermore, the actual empirical evidence for this perspective is somewhat sparse, as studies typically are case studies of single locations [25]. Although there is some evidence that characteristics of the physical form impact social interaction among residents[25], there is less evidence that these characteristics affect a sense of place and feelings of attachment [25]. For example, a study of elderly residents in Aichi, Japan tested and found no relationship between neighborhood walkability scores and reported social capital [31]. A study of Dutch neighborhoods found that although the presence of more meeting places increased reported stimulation, it had no effect on reported cohesion [32].

To consider why the meso- or macro- scale of physical features might be important, it is necessary to consider the spatial extent of residents' social networks. There is a relatively well established literature showing that residents tend to form social ties with others based on a distance decay function [4]–[7], [33], [34]. That is, residents are most likely to form ties to those living near them, and this likelihood drops sharply when moving further away from the residence. Indeed, studies have shown that there are differences in social tie formation even within a very short physical distance [4]–[6]. Other research has suggested that these distance decay functions also operate on much larger scales [7]. That is, the particular distance decay function does not just hold for shorter distances of a few kilometers, but can be observed for the presence of ties 100 s of kilometers away from a resident. A key implication is that if neighborhood social ties indeed are formed based on a particular physical distance decay function, then the physical features of the meso-environment of the neighborhood may have important consequences in that they impact the number of potential ties that a resident might be able to form, particularly at the nearer end of the distance decay function.

We suggest that there are two key characteristics of the physical environment that can have important consequences for the formation of social ties, and hence residents' sense of cohesion which are not currently considered in research. We term these two characteristics 1) *social holes*; and 2) *wedges*, each of which is discussed in turn below. First, whereas much of the city landscape is characterized by the presence of residential housing units, there are parts of the landscape that contain “holes” where there is a limited residential population. If social ties are formed based on a particular physical distance decay function, then the presence of such social holes nearby to residents would reduce the potential number of ties within a particular mid-range distance of a neighborhood. By mid-range, we are referring to the area beyond one's street block and immediately adjacent blocks up to about 3 miles (approximately 5 kilometers). This could then reduce the number of social ties residents will have, the amount of neighboring they can do, and their sense of cohesion with the neighborhood.

Two particularly notable social holes in the landscape are industrial areas and parks, yet these features differ somewhat. On the one hand, parks have the potential to be a gathering spot for the neighborhood, particularly during weekends and holiday periods. This implies that parks may provide opportunities to socially engage with fellow residents during the daytime. On the other hand, there is evidence that the density of crime occurring in or near parks is substantially higher than crime occurring across a given area [35]. As a consequence, evidence suggests that residents may avoid some parks out of fear of victimization [36]. In either case, the presence of a park additionally reduces the number of persons residing within a mid-distance radius of a household, simply because this creates a social hole in which no residents are located. That is, a household with a park nearby will have fewer nearby households with which to interact than a household surrounded by all housing units. In contrast, industrial areas provide no such potential gathering component: although they experience an influx of workers during working hours, they typically do not draw non-workers to them and are typically nearly completely empty at night and on weekend. Thus industrial areas represent purer *social holes*.

Second, whereas social ties are influenced by a distance decay function, certain features of the environment can act as wedges that make it difficult to form social ties even among residents who are, at least spatially, proximal to each other. These features thus act as a physical boundary. The question then is the *degree of permeability* in the boundary caused by a particular feature. That is, how much *social porosity* is there across the boundary? Rivers and large highways are examples of two such wedges. Rivers are particularly impermeable, and crossing them typically requires a bridge or a ferry connection. Households that live near a river can only easily cross it on foot if they are near a bridge; otherwise, passage will be extremely difficult.

In contrast, highways, at least in some instances, may be more permeable than rivers. Nonetheless, if the highway is large enough, it may be that the only way to cross it will be at points where there are over- or under-passes. In such instances, the permeability of a highway could be nearly as low as a river.

The present study examines the effect of wedges and social holes on the level of neighboring, social cohesion and neighborhood attachment. Controlling for well-known features of the neighborhood that impact on ties and social cohesion, we assess the level of *social porosity* in the presence of wedges and social holes of differing spatial scales. Drawing on the Australian Bureau of Statistics (ABS) census data, topographic data and the Australian Community Capacity Study (ACCS) survey data we describe how the four key environmental features of rivers, highways, industrial areas, and parks could impact upon neighborhood sociability. This paper addresses three key questions:

1. What is the spatial distribution of socially impeding environmental features (e.g. rivers and industrial areas) across urban residential areas?; and,
2. To what extent do these features fragment these areas? ; and,
3. To what extent does the degree of neighborhood fragmentation impact general neighboring, cohesion and neighborhood attachment?

## Data and Study Location

### Data

**Figure 1:**
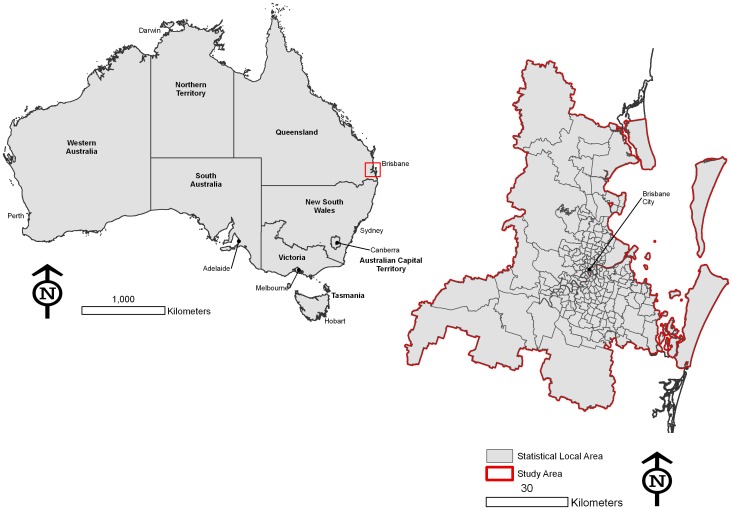
The Brisbane Statistical Division, Australia.

This paper draws on several data sources. The survey data are derived from the Australian Community Capacity Study (ACCS). The ACCS is a longitudinal panel study of urban communities in Australia that is supported by Australia Research Council funding [37]–[39]. The overarching goal of the ACCS is to understand and analyze the key social processes associated with the spatial variation of crime and disorder across urban communities over time. This study employs data collected in 2010 representing Wave 3 of the ACCS survey in the Brisbane Statistical Division (BSD) located in Queensland. Brisbane is the state capital of Queensland and the third largest city in Australia with a population of approximately 1.9 million people. This area is shown in the map in Figure 1. The Brisbane ACCS sample comprises 148 randomly drawn state neighborhoods (suburbs) with a residential population ranging from 245 to 20,999 (total suburbs in the BSD = 429 with a residential population ranging from 15 to 21,001). Note that In Australia, the term “suburb” is used to refer to a feature that in the U.S. would be referred to as a “neighbourhood”. Suburbs are similar to census tracts in the U.S. context, though in some cases Brisbane suburbs may be larger than census tracts as they are not determined by population. Throughout, we use the more familiar term “neighbourhood” to refer to these. Residents provided their address details, and each respondent was geocoded to a point location. This information is used to derive the various metrics described shortly.

For the Wave 3 Brisbane ACCS survey sample (N = 4,404 comprising 2,248 longitudinal and 2,156 top up participants), respondents were randomly selected using random digit dialing [38]. We were able to geocode 4,351 of the respondents to neighborhoods and therefore they constitute the study sample. The overall consent and completion rate for the Brisbane ACCS Wave 3 was 68.5 percent; for further information see [38]. This rate represents the number of interviews completed proportional to the number of in scope contacts. The Brisbane ACCS panel survey sample comprises respondents from the two previous waves and top-up respondents randomly selected in Wave 3. As there is attrition in the longitudinal sample, in order to maintain ecometrically valid indicators of social processes [40], a top up sample is generated at each wave. The number of residents needed to maintain ecometric reliability is assessed using power analyses for multilevel samples.

The ACCS survey was conducted from 25^th^ August to 15^th^ December 2010 by the Institute for Social Science Research at the University of Queensland. Trained interviewers utilized computer-assisted telephone interviewing to administer the survey which lasted approximately 25 minutes. The in-scope survey population comprised all people aged 18 years or over who were usually resident in private dwellings with telephones in the selected neighborhoods in Brisbane.

In addition to the survey data, we also utilized census data from the 2006 Australian Bureau of Statistics (ABS). We then merged these census and survey data information with the physical environment of the region. These data included information on the road network, parkland, industrial areas and rivers. We integrated these spatial data sources using Geographic Information System (GIS)-based procedures to provide the necessary base data. These data included a MapInfo Street Pro database of the road network coupled with the Digital Cadastral Database (DCDB) and Queensland Valuation and Sales (QVAS) data depicting land use (i.e. parkland and industrial areas) and the spatial scale of the individual land parcel.

#### Dependent Variables

There are three dependent variables for the analyses. The three variables are indices that were combined through maximum likelihood exploratory factor analysis, and then computing factor scores. The first outcome is a measure of *neighboring*. This index combined three items measuring the frequency of neighborly exchange, and has a Cronbach's alpha of .77. The items that comprise this index ask residents to comment on how often you and people in your community 1) do favors for each other; 2) visit in each other's homes or on the street; and 3) ask each other advice about personal things such as child rearing or job openings. This index has been used in previous research [41] and depicts what Woldoff [42: 97] classifies “more intense neighbor relationships” or what Sampson [43: 19] refers to as the “activation” of an individual's neighborhood ties. The second outcome variable is a measure of *attachment to the community*. This measure asks respondents to report on their own sense of attachment to the community, and is constructed by combining three questions: 1) I feel that I belong to this local community; 2) I would like to be living in this local community in three years; 3) I am proud to live in this local community. The Cronbach's alpha is .82. The third outcome variable is a measure of *perceived cohesion in the community*. This measure asks respondents to report on their perceptions of the general sense of cohesion in the community as felt by others living there, and is constructed by combining four questions: 1) People in this community are willing to help their neighbors; 2) This is a close-knit community; 3) People in this community can be trusted; 4) People in this community do not share the same values (reverse coded). The Cronbach's alpha is .71.

### Independent variables

The key independent variables in the analyses measure the features of the physical environment of interest to this paper. For all of these physical characteristics, there is not necessarily one obvious way to measure them and their impact on sociability. Therefore, below we discuss different possible measures we might construct, and what they capture conceptually. We constructed such measures and compared their differing effects in the models presented. Two of our features capture “holes” in the social environment: i.e. parks and industrial areas. For each of these features we constructed two measures. The first captures the density of the feature (parks or industrial areas) in relation to the overall size of a neighborhood (i.e. the area within a neighborhood). The second measure captures the distance of a neighborhood to their nearest park or industrial area. These distance measures are constructed by first computing the distance of each sampled household in a neighborhood to the nearest industrial area (or park), and then computing the mean of these distances for all individuals in the neighborhood. Given the distance decay function typically observed for social tie formation, we would expect that closer residence to one of these social holes would reduce the level of neighboring, attachment, and cohesion. In ancillary models, we also included household-level measures of distance to specific environmental features (e.g. an industrial area). These measures never added additional information beyond those constructed computing the mean for the entire neighborhood. Thus, this appears to be a neighborhood-level effect, and not an individual-level one. A counter-hypothesis is that parks not only constitute social holes, but also are social gathering spots and therefore might have a positive effect on tie formation. We are able to assess these competing hypotheses here.

The other two features we study capture “wedges” in the social environment. These two features are highways and rivers. We constructed five different measures to capture possible wedge effects. For both highways and rivers we computed: 1) the number of *fragments* in the neighborhood induced by the feature. In other words, given the hypothetical situation where a single highway divides a neighborhood in half then the number of fragments created would be 2; 2) the *patch density* of the feature in the neighborhood (defined as the number of individual fragments of a particular type divided by the total area of the neighborhood); 3) the *total length* of the feature in the neighborhood; 4) the *density* of the feature within a neighborhood (the total area of the feature in the neighborhood divided by the total area of the neighborhood); and 5) the *average distance* to the feature for residents in the neighborhood. One of the neighborhoods was an outlier for the measure of length of a river; we therefore created an indicator variable for this neighborhood and included it in all models that included the river length measure.

To develop the aforementioned measures further we next discuss what each captures conceptually, particularly in how they capture the effects of highways and rivers (which can differ in their level of permeability). First, the *fragments* variable captures the number of sub-area fragments of a neighborhood induced by the feature (i.e. a highway or river). It is hypothesized that each additional fragment will reduce neighboring and cohesion, and that this measure should have a stronger effect for more impermeable physical boundaries (i.e., rivers). Second, the *patch density* variable standardizes the fragments measure by the total area of the neighborhood. Thus, this measure tests whether or not it is the *size* of the fragments that matter most for neighboring, cohesion and neighborhood attachment. Third, the *length* of the feature in a neighborhood captures the extent to which the neighborhood is split by the feature. This will likely be particularly important for a relatively impermeable boundary, such as a river. Thus, for a neighborhood in which a river is only present for a short distance, it will only cut off the edge of the neighborhood (and the few households on the other side of the river). However, a river that is in a neighborhood for a long distance is more often splitting the neighborhood in two, and may have a strong effect on neighboring and cohesion. A highway that splits a neighborhood in two may not have as much effect on social relations, given that it is a more permeable boundary. Fourth, the *density* of the feature in the neighborhood standardizes the area of the feature (i.e, the length variable) by the total area of the neighborhood. This measure does not simply assume that it is the area of a feature in a neighborhood that matters, but rather that it is the proportion of the total area impacted by the feature that is important. It is posited that a high proportion of area fragmentation generated through the presence of features in a neighborhood will decrease social porosity within the locale. Fifth, the *average distance* of the neighborhood from a feature captures the average proximity of residents to the features in the neighborhood. It is assumed that closer residence to one of these physical features results in increased social fragmentation created by such features.

#### Neighborhood-level control measures

To minimize the possibility of detecting spurious results for the effects of the physical environment on neighboring, attachment, and cohesion, we included several measures capturing the social characteristics of the local area from the ABS 2006 census data, and several socio-demographic household measures previously found to explain levels of neighboring and cohesion, as described earlier. We discuss each in turn below.

*Residential stability* is measured as the proportion of people living at a different address 5 years prior, from the ABS 2006 census data. *Median income* is computed from the same source, and captures the socio-economic status of the local area. Studies in the U.K. demonstrate that individuals living in diverse communities know fewer neighbors and speak to them less frequency [44]. In Australia, this relationship is also found, though natives are statistically more likely to ‘hunker” than non-natives [45]. We therefore constructed a measure of the *percent perceived non-Anglo* based on the responses of residents to the ACCS. Finally, given that the density of the local population likely impacts the possibility of social interactions [7], we constructed a measure of *population density* as the total persons per square kilometer.

#### Household level measures

We constructed several household-level measures capturing socio-demographic characteristics. We constructed measures of approximate annual household income (1 =  less than $20, 000, 2 = $20, 000 to $39,999; 3 = $40, 000 to $59,999; 4 = $60,000 to $79,999; 5 = $80,000 to $99,999; 6 = $100,000 to $119,999; 7 = $120,000 to $149,999; 8 = $150,000 or more); highest level of education (1 =  post graduate qualifications; 2 =  a university or college degree; 3 =  a trade, technical certificate or diploma; 4 =  completed senior high school; 5 =  completed junior high school; 6 =  primary school; 7 =  no schooling; 8 =  other response); whether own or rent; length of residence at the current address (1 =  less than 6 months; 2 =  6 months to less than 12 months; 3 =  12 months to less than 2 years; 4 =  2 years to less than 5 years; 5 =  5 years to less than 10 years; 6 =  10 years to less than 20 years; 7 =  20 years or more); whether the respondent speaks a language other than English at home and whether the respondent has dependent children. We included measures of marital status (single, widowed, or divorced, with married as the reference category), age and gender. We constructed several ancestry measures: 1) Middle Eastern; 2) Asian; 3) South-Eastern European; 4) South African; 5) Aboriginal and Torres Strait Islander. Northern Europeans are the reference category. The summary statistics for the variables included in the analyses are displayed in Table 1.

**Table 1:** Summary statistics of variables used in analyses.

*Outcome variables*	Mean	Std Dev
Neighboring	−0.007	0.906
Attachment to neighborhood	0.001	0.926
Cohesion in neighborhood	−0.004	0.891
***Physical characteristics of neighborhood***		
*Park*		
Proportion of neighborhood	0.078	0.104
Average distance	0.540	0.700
*Industrial areas*		
Proportion of neighborhood	0.018	0.039
Average distance	2.181	2.550
*River*		
Number of splits	1.556	0.880
Density of neighborhood	0.082	0.106
Patch proportion of neighborhood	0.139	0.138
Length	0.017	0.075
*Highways*	0.168	0.374
Number of splits	1.184	0.427
Density of neighborhood	0.295	0.203
Patch proportion of neighborhood	0.136	0.144
Length	0.177	0.166
***Social characteristics of neighborhood***		
Residential stability	−0.028	0.648
Median income	1.243	0.375
Percent non-Anglo	23.252	11.570
Population density	1.377	1.168
***Household level measures***		
Speak only English at home	88.9%	
Owner	85.3%	
Single	12.3%	
Widowed	6.6%	
Divorced	9.1%	
Married	72.1%	
Female	59.0%	
Have children	75.3%	
Middle eastern	1.7%	
Asian	5.9%	
Southern European	3.9%	
African	0.7%	
Indigenous	0.8%	
White	87.0%	
Education	3.744	1.388
Household income	4.378	2.159
Length of residence	5.403	1.345
Age	0.512	0.152

Note: Sample size is 4,351 respondents in 146 neighborhoods.

## Methods

Given that we have households nested within neighbourhoods, we estimated multilevel linear models in which the outcome variable is each individual's report of the level of neighboring or cohesion in the neighbourhood, or their own reported attachment to the neighbourhood. The models are estimated as: 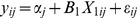
(1)

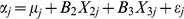
(2)where *y_ij_* is the construct of interest (for example, perceived cohesion) reported by individual *i* in neighborhood *j*, X_1*ij*_ is a vector of individual-level demographic characteristics whose effects on the outcome measure are captured in the Β*_1_* vector, and *ε_ij_* is a disturbance with an assumed normal distribution. The *α_j_* is a random intercept that represents the neighbourhood level latent variable of the various constructs (for example, perceived cohesion) and is the outcome variable in equation 2. In equation 2, X_2*j*_ is a vector of the neighbourhood physical characteristics (as described above) whose effects are contained in the Β*_2_* vector, X_3*j*_ is a vector of neighborhood-level socio-demographic measures with effects contained in the Β*_3_* vector, and *ε_j_* is a normally distributed disturbance. The coefficients in the Β*_2_* vector are the crucial tests. By estimating these coefficients separately, we will be able to assess the relative strength of these various measures of “holes” or “wedges” in the social environment.

Our strategy is to first estimate models in which we include the measures of physical characteristics one at a time to assess the effect of these various possible measures on neighboring, attachment, and cohesion. The models always control for all the household and socio-demographic neighborhood measures described in the data section. Following that, we will estimate models which simultaneously include a single measure of each of these four features (rivers, highways, industrial areas, parks). In these latter models, we include the measure of each physical feature which exhibited the strongest effect in the initial models. We tested for nonlinear effects for all measures by constructing polynomial versions of all of the continuous measures; we report the nonlinear results in instances in which they were significant. This research was approved by the University of Queensland Institutional Review Board.

## Results

In the first set of models presented in Table 2, we assess the impact of various neighborhood physical features on the level of neighboring among residents (these models control for all the household and socio-demographic neighborhood measures described in the data section). When assessing the impact of “social holes”, measuring the density of the neighborhood constituted by these features is not an important predictor of neighboring. Instead, it is the *distance* of the neighborhood from a particular feature that matters. Neighborhoods that are closer to industrial areas or parks have less neighboring, even controlling for several household-level measures as well as key measures capturing the social composition of the neighborhood. A one standard deviation increase in distance from a park (0.7 kilometers) increases neighboring .056 standard deviations (β = .056), whereas a one standard deviation increase in distance from an industrial area (2.55 kilometers) increases neighboring .082 standard deviations (β = .082).

**Table 2:** Models with neighboring as an outcome.

(1)	(2)	(3)	(4)	
Park density	Distance to park	Industrial density	Distance to industrial	
0.104	0.073**	0.085	0.029**	
(0.65)	(2.98)	(0.20)	(4.38)	
(5)	(6)	(7)	(8)	(9)
River fragments	River density	River length	River patches	Distance to river
−0.021	−0.327*	−2.15**	−0.063	−0.012
−(1.11)	−(2.11)	−(2.81)	−(0.52)	−(0.50)
(10)	(11)	(12)	(13)	(14)
Highway fragments	Highway density	Highway length	Highway patches	Distance to Highway
−0.078*	0.044	0.056	0.000	−0.001
−(2.01)	(0.54)	(0.57)	(0.00)	−(0.04)

**p<.01(two-tail test),

*p<.05 (two-tail test). T-values in parentheses. Multilevel models with clustering based on neighborhood.

Turning to the measures capturing wedges, we first focus on those measuring rivers. The measures of *density* of a river in a neighborhood and the *length* of a river in a neighborhood have the strongest negative impacts on neighboring. A one standard deviation increase in the density of a river in the neighborhood reduces neighboring 0.4 standard deviations (β = −.038). However, the length of the river in the neighborhood has a particularly powerful impact as a one standard deviation increase in river length (0.7 kilometers) in the neighborhood reduces neighboring .18 standard deviations (β = −.177). The other techniques used to measure the presence of a river do not significantly impact neighboring: i.e., the number of river fragments, the number of river patches, and the average distance to a river. Of the measures capturing the impact of a highway, it is the number of *fragments* in the neighborhood created by the highway that has the strongest negative effect on neighboring. Each additional fragment in the neighborhood created by highways reduces neighboring 0.09 standard deviations (β = −0.086). The other measures of highways do not affect neighboring: i.e. highway density, the number of highway patches, the length of the highway, or the average distance to a highway.

We next asked whether these physical features impacted residents' sense of attachment to, or perceived cohesion in, the neighborhood. These results are presented in Tables 3 and 4 and we discuss them simultaneously given their similarity. Two features of social holes (parks and industrial areas) have similar impacts on attachment and cohesion as they did for neighboring. The average distance from a feature for neighborhood residents increases attachment and cohesion. Thus, residents living in neighborhoods adjacent to a park (β = .08) or an industrial area (β = .072) report the lowest sense of attachment to the neighborhood, whereas those living further away from such features report higher levels of attachment, even controlling for these various household-level measures as well as key measures of the social composition of the neighborhood. And neighborhoods that are adjacent to industrial areas (β = .109) or parks (β = .094) report the lowest levels of cohesion. The density of a feature in the neighborhood does not impact neighborhood attachment or cohesion.

**Table 3:** Models with attachment to neighborhood as an outcome.

(1)	(2)	(3)	(4)	
Park density	Distance to park	Industrial density	Distance to industrial	
0.181	0.106**	−0.659	0.026**	
(0.91)	(3.62)	−(1.27)	(3.12)	
(5)	(6)	(7)	(8)	(9)
River fragments	River density	River length	River patches	Distance to river
−0.014	−0.266	−1.916*	−0.127	0.043
−(0.60)	−(1.39)	−(2.03)	−(0.84)	(1.41)
(10)	(11)	(12)	(13)	(14)
Highway fragments	Highway density	Highway length	Highway patches	Distance to Highway
−0.121*	−0.108	−0.114	−0.114	0.033
−(2.49)	−(1.06)	−(0.93)	−(0.82)	(0.90)

** *p<.01(two-tail test)*,

* *p<.05 (two-tail test). T-values in parentheses. Multilevel models with clustering based on neighborhood.*

**Table 4:** Models with neighborhood cohesion as an outcome.

(1)	(2)	(3)	(4)		
Park density	Distance to park	Industrial density	Distance to industrial		
0.068	0.12**	−0.436	0.038**		
(0.36)	(4.45)	−(0.89)	(5.18)		
(5)	(6)	(7)	(8)		(9)
River fragments	River density	River length	River patches		Distance to river
0.002	−0.354*	−1.8*	0.088		0.006
(0.08)	−(2.01)	−(2.06)	(0.62)		(0.22)
(10)	(11)	(12)	(13)		(14)
Highway fragments	Highway density	Highway length	Highway patches		Distance to Highway
−0.08†	0.083	0.076	0.092		0.015
−(1.76)	(0.88)	(0.66)	(0.71)		(0.44)

**p<.01(two-tail test),

*p<.05 (two-tail test),

† *p<.05 (one-tail test). T-values in parentheses. Multilevel models with clustering based on neighborhood.*

For the wedge features (rivers and highways), the impact on attachment and cohesion is similar to the impact on neighboring. Among the measures capturing the impact of a river, the length of the river has the strongest effect. Thus, each one standard deviation increase in the length of a river in the neighborhood decreases residents' sense of attachment and perceived cohesion with the neighborhood .15 standard deviations (β = −.154 and β = −.151, respectively). The measure of river *density* has a significant negative effect on cohesion (β = −.042), but it is weaker than the measure of river length. The other measures capturing the effect of a river on a neighborhood are not related to sense of attachment. Among the measures capturing the impact of highways, the number of fragments in a neighborhood created by highways has the strongest negative impact on sense of attachment (β = −.131) and perceived cohesion (β = −.09). The other measures of highway impact are not statistically significant.

### Models including all four physical features simultaneously

**Figure 2:**
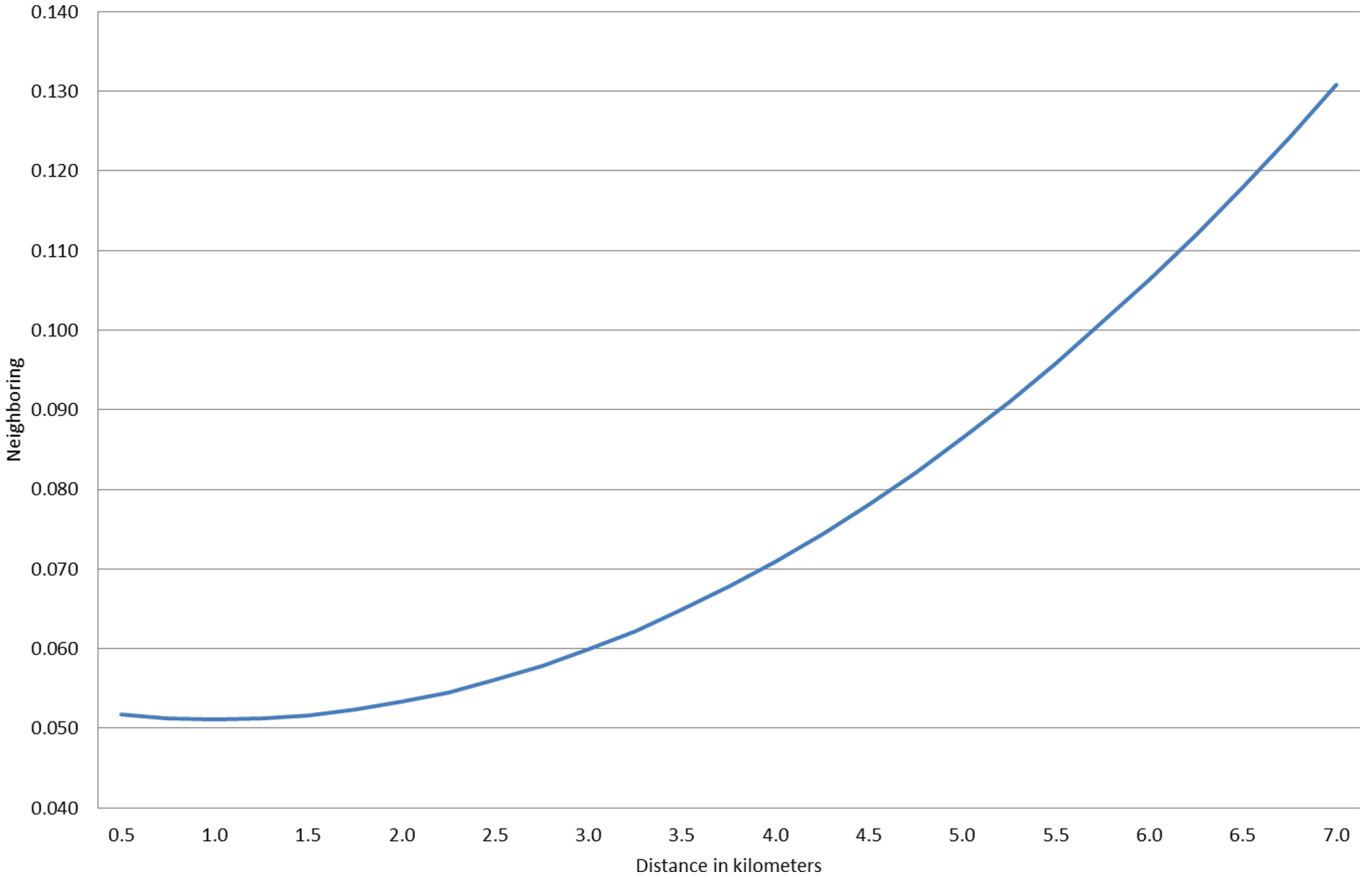
Effect of distance (kilometers) from industrial area on neighboring.

We next estimated models that included all four physical features simultaneously and the results are displayed in Table 5. For these models, we kept the strongest measure of each physical characteristic construct from the previous models. In model 1 with neighboring as an outcome, we see that of the two constructs capturing holes in the social environment, it is distance to an industrial area that has the stronger impact on neighboring, even controlling for the wedge measures. We tested and found a nonlinear effect, and it is plotted in Figure 2: the left side of the figure shows that the lowest levels of neighboring occur in neighborhoods that are adjacent to an industrial area. However, the level of neighboring increases nonlinearly as the neighborhood is further away from an industrial area (the right hand side of this figure). A neighborhood 6.5 kilometers (approximately, 4 miles) from the nearest industrial area has 0.07 standard deviations more neighboring than a neighborhood that is immediately adjacent to an industrial area. The effect of distance to a park on neighboring is not statistically significant in this model accounting for these other physical features.

**Table 5:** Full models predicting neighboring, attachment to neighborhood, and cohesion in neighborhood.

	**Neighboring**	**Attachment to neighborhood**	**Cohesion in neighborhood**
***Neighborhood measures: physical characteristics***			
Distance to industry	−0.005	0.011	−0.011
	−(0.25)	(1.09)	−(0.56)
Distance to industry squared	0.002†		0.003*
	(1.86)		(2.13)
Distance to park	0.014	0.075*	0.056†
	(0.45)	(2.06)	(1.70)
Distance to park squared			
Highway fragments in neighborhood	−0.041	−0.081†	−0.030
	−(1.13)	−(1.74)	−(0.73)
River length in neighborhood	−2.186**	−1.818*	−1.908*
	−(2.99)	−(2.00)	−(2.37)
Very large value of river length	1.983**	1.310	1.623†
	(2.65)	(1.40)	(1.96)
***Neighborhood measures: social characteristics***			
Residential stability	0.008	0.011	0.093**
	(0.29)	(0.37)	(3.15)
Median income	−0.003	0.230**	0.205**
	−(0.06)	(4.37)	(4.20)
Percent non-Anglo	−0.007**	−0.009**	−0.009**
	−(4.31)	−(4.67)	−(4.71)
Population density	−0.004	0.004	−0.003
	−(0.27)	(0.23)	−(0.18)
***Individual and household measures***			
Speak only English at home	0.009	−0.145*	−0.029
	(0.16)	−(2.43)	−(0.51)
Education	0.030**	−0.005	0.003
	(2.77)	−(0.45)	(0.27)
Household income	0.017†	0.014	0.017†
	(1.84)	(1.45)	(1.96)
Owner	0.108*	0.112*	0.086*
	(2.46)	(2.55)	(2.02)
Length of residence	0.033**	0.042**	0.001
	(2.76)	(3.53)	(0.09)
Single	−0.077	−0.115*	−0.054
	−(1.56)	−(2.25)	−(1.13)
Widowed	0.103†	−0.014	0.062
	(1.68)	−(0.22)	(1.08)
Divorced	−0.048	−0.130**	−0.085†
	−(0.96)	−(2.60)	−(1.77)
Age	−0.050	0.572**	0.434**
	−(0.36)	(4.11)	(3.28)
Age squared		−1.710**	
		−(3.14)	
Female	0.069*	0.080**	0.105**
	(2.50)	(2.89)	(3.91)
Have children	0.079**	0.030*	0.040**
	(5.67)	(2.18)	(2.97)
Middle eastern	−0.527**	−0.143	−0.128
	−(4.24)	−(1.14)	−(1.02)
Asian	−0.265**	0.017	0.065
	−(3.52)	(0.22)	(0.88)
Southern European	−0.111	−0.032	0.007
	−(1.57)	−(0.45)	(0.10)
African	−0.381*	−0.044	−0.323†
	−(2.31)	−(0.27)	−(1.95)
Indigenous	−0.151	0.114	0.090
	−(1.02)	(0.78)	(0.64)
Intercept	0.053	0.103	0.024
	(0.99)	(1.59)	(0.39)

**p<.01(two-tail test),

*p<.05 (two-tail test),

† *p<.05 (one-tail test). T-values in parentheses. Multilevel models with clustering based on collection district.*

We see in this same model that of the two wedge constructs, the presence of a river has a stronger impact on neighboring behaviour than does the presence of a highway. Thus, a one standard deviation increase in length of river in the neighbourhood reduces the level of neighboring 0.18 standard deviations. The effect of highway fragments is not statistically significant in this model that takes into account these other physical features of the environment. When we substituted the measure of river density for the measure of length of river edges in each of the models displayed in Table 5, we found that the density measure had a significant negative effect on neighboring and cohesion, but had a negative, but not statistically significant, effect on neighborhood attachment. For all three outcomes, the overall model fit was superior when using the length of river edges measure, rather than the river density measure. We therefore only present the results using the river edge length measure.

In model 2, in which the outcome is attachment to the neighbourhood, we see that of the two constructs capturing holes in the social environment, the presence of a nearby park, rather than a nearby industrial area, most strongly impacts attachment to the neighborhood. A one standard deviation increase in distance from a park (0.7 kilometre or 0.4 miles) increases the level of neighbourhood attachment 0.06 standard deviations. In this model, both wedge features of the environment reduce sense of attachment. Each additional fragment created by the presence of highways reduces attachment 0.09 standard deviations. And increasing the length of a river in the neighbourhood 0.7 kilometers (one standard deviation) reduces attachment 0.15 standard deviations.

**Figure 3:**
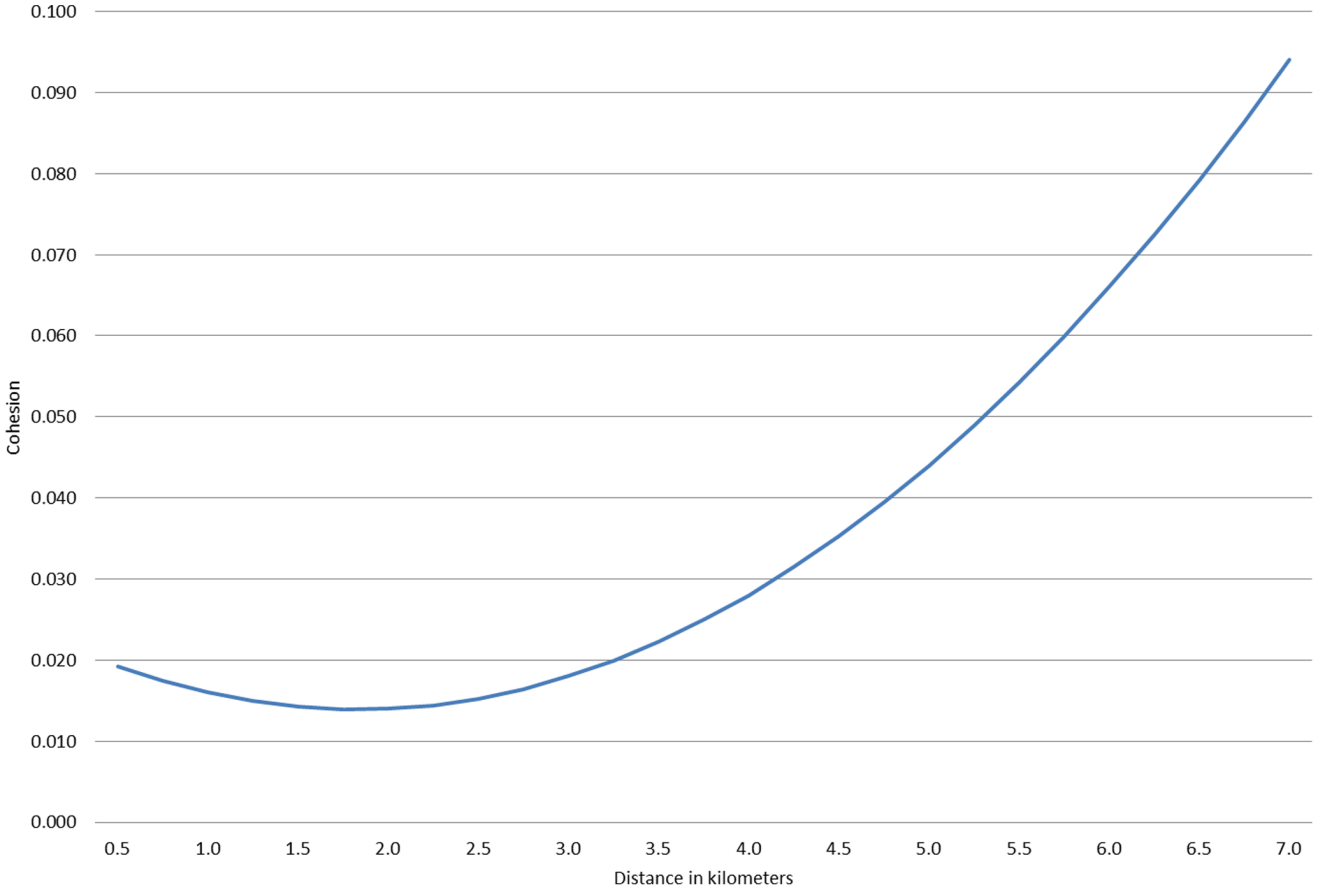
Effect of distance (kilometers) from industrial area on cohesion.

In model 3, in which the outcome is perceptions of cohesion in the neighbourhood, both measures capturing holes in the social environment appear important. There is a modest effect in which neighbourhoods closer to a park report less cohesion. A stronger, nonlinear, effect is observed for neighbourhoods that are near an industrial area. This effect is plotted in Figure 3, and shows that neighbourhoods from 0 to 3 kilometres of an industrial area routinely report lower levels of cohesion. However, beyond this distance there is a sharp nonlinear increase in reported cohesion. A neighborhood that is 6.5 kilometres from an industrial area reports 0.05 standard deviations more cohesion than one 3 kilometres (approximately 1.9 miles) from an industrial area. Although this shape is slightly u-shaped on the left side of this graph, the differences among these low values are not statistically significant, and thus this line is essentially flat in this range. In this same model we see that each additional 0.7 kilometers of a river's length in the neighborhood reduces the sense of cohesion .16 standard deviations. More fragments in the neighborhood created by highways do not impact cohesion once taking into account these other features of the model.

Finally, we briefly discuss the results of our control variables, which are generally in the expected direction. For the neighborhood measures capturing the social environment, the presence of a higher percentage non-Anglo has a negative relationship with all three of these outcomes. That is, social cohesion, attachment and neighboring are lower in neighborhoods where residents perceive greater numbers of non-white residents; see also [44]–[48] Neighborhoods with higher median income have more cohesion and attachment, but do not experience higher levels of neighboring. Neighborhoods with greater residential stability have a higher sense of cohesion, but do not neighbor more or feel more strongly attached to their neighborhood. Among the household measures, owners, females, and those with children all report higher levels of all three outcome measures. Those with longer length of residence report more neighboring and attachment, but not any more cohesion. Although age does not affect neighboring, it does increase perceptions of cohesion and attachment (though the latter outcome exhibits an inflection point at higher ages). And while some of the ethnic measures are associated with less neighboring, they are generally not related to cohesion or attachment.

## Discussion

This study has demonstrated that physical features of the environment have important consequences for the general sense of neighboring, attachment, and cohesion in neighborhoods. We have suggested that physical features can be thought of in two key ways: as *holes* and as *wedges* in the social environment. We utilized key measures from the geography literature to assess how these features carve up the neighborhood and capture their effect on neighborhood sociability and attachment. These measures of the nodes and wedges created by the physical environment of the neighborhood showed robust negative effects on cohesion and neighboring, even when controlling for individual and household characteristics, as well as social characteristics of the neighborhood.

We measured two types of wedges in the physical environment (rivers and highways), and found that they impacted social relations and subsequent perceptions of cohesion and attachment. We found that the strongest impact of a wedge occurred when measuring the length of a river within a neighborhood. Given that rivers provide a strong boundary, a neighborhood with a long stretch of river is arguably split into separate areas, which has consequences for social relations. In such neighborhoods, it is difficult to establish ties with residents on the other side of the river, which decreases the level of neighboring, attachment, and cohesiveness. This implies that there is relatively little social porosity across a river.

The wedges created by highways had the strongest negative effect on social relations when measured by the number of fragments they created in the neighborhood. Given that a highway is somewhat more permeable than a river, a long highway splitting a neighborhood likely does not as strongly impact social relations. Instead, it is when numerous highways create *smaller fragments* of areas within a neighborhood that we observed a reduction in neighboring, attachment, and cohesion. Although highways are more permeable than rivers, it still is the case that by splitting a neighborhood into a greater number of subareas (and possibly cliques of social ties), highways can impact residents' perceptions of cohesion. Ties may not be entirely fragmented by a single highway, but the presence of several appears to have a stronger effect on residents' perceptions.

**Figure 4:**
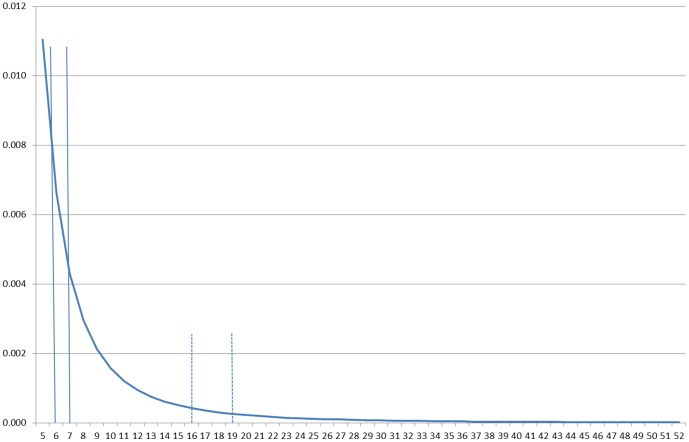
Probability of tie based on hypothetical spatial interaction function.

We also hypothesized and found that holes in the social environment led to a reduced sense of cohesiveness and attachment to the neighborhood. We measured two types of social holes–industrial areas and parks–and found that both impacted social relations. Given that social ties typically form based on a distance decay function, it is not surprising that it was the *nearness* of these social holes, and not their relative size in the neighborhood, that impacted social relations. This implies that the distance decay of ties is quite important, as the closeness of the feature captures a higher point on the distance decay curve [7]. To understand this, in Figure 4 we display a hypothetical spatial interaction function (SIF) which displays the probability of interaction based on Festinger's results as estimated by Butts et al [7]. This has a general power law form with the SIF declining approximately with distance (*d*) raised to −2.8. Note that a relatively close social hole with an area of one unit from 6 to 7 on this function (the solid lines) implies that the probability of interaction with residents who might have lived in this area is .0043, whereas a further away social hole with an area of three units from 16 to 19 on this function (the dashed lines) implies that the probability of interaction with residents who might have lived in this area is .0009. This is an expected implication from such a function, and importantly our findings are consistent with this expectation.

A second important consequence of social holes is that they appeared to impact the general social relations within a neighborhood, and not just the perceptions of specific individuals. Thus, the closeness of a neighborhood to a park or industrial area exhibited a neighborhood level effect, as there was no evidence that an individual's own distance to the physical feature had an additional effect on these outcomes. Thus, social holes appear to impact the general pattern of social relations in a neighborhood, as well as the general sense of attachment to, or cohesiveness in, the neighborhood.

We acknowledge some limitations of this study. The first concerns the measurement of fragmentation at a neighborhood level given that we know the spatial distribution of population to be non-uniform across a given neighborhood. In the current implementation of the metric this non-uniform population distribution was not explicitly accounted for in the analysis given constraints in size of the census reporting units despite this study drawing upon the most detailed spatial data available to compute the various measures (i.e. the small census geography and parcel-based land use information). To address this issue would require a remote sensing-based approach to identify locales of populations within the finest geography of census units (i.e census collection districts) and then compute the metrics on these newly demarcated spatial units. The second limitation relates to the homogenous treatment of features, in other words one park is considered the same in its implication for social fragmentation as the next; as such the functional attributes of specific features were not considered. Given that parks differ in their potential for impacting sociability, teasing apart the heterogeneous effects for parks would require more detailed survey data to more comprehensively assess the role of parks, their design and characteristics in relation to usage and their role within the neighborhood.

## Conclusion

Despite these limitations, a key contribution of this study is demonstrating the importance of the physical environment at a more meso-scale on social relations. We have demonstrated that the physical environment has notable impacts on neighboring and perceptions of cohesion. We found that both parks and industrial areas serve as social holes that impact neighboring and cohesion when measuring the physical distance of these features to the neighborhood. We acknowledge that these are not the only possible constructs that might be used to measure *social holes*; other candidates include areas such as office or retail areas. Nonetheless, we believe this study is an important first step in assessing the importance of social holes, and an important future direction is for studies to test the impact of other social holes on social relations. We also found that both rivers and highways serve as wedges that impact neighboring and cohesion, although each effect is best captured in different manners. On the one hand, highways most strongly reduced neighboring and cohesion when measured as the number of fragments they created in a neighborhood. Thus, each fragment presumably contains people who are less likely to be attached to residents in the other fragments. On the other hand, the strong negative effect of rivers on sociability was detected when measuring the length of the river in the neighborhood; this arguably is a relatively impermeable boundary, and the more it isolates households from each other, the more it will reduce neighboring and cohesion. The physical environment impacts residents' social behavior and has important consequences for the level of neighboring and cohesion within neighborhoods.

## References

[1] Sampson RJ (1988) Local Friendship Ties and Community Attachment in Mass Society: A Multilevel Systemic Model. American Sociological Review 53: 766–779.

[2] Bolan M (1997) The Mobility Experience and Neighborhood Attachment. Demography 34: 225–237. 9169279

[3] Mesch GS, Manor O (1998) Social Ties, Environmental Perception, and Local Attachment. Environment and Behavior 30: 504–519.

[4] Festinger L, Schachter S, Back K (1950) Social Pressures in Informal Groups. StanfordCA: Stanford University Press. 197 p.

[5] Hipp JR, Perrin AJ (2009) The Simultaneous Effect of Social Distance and Physical Distance on the Formation of Neighborhood Ties. City & Community 8: 5–25.

[6] Caplow T, Forman R (1950) Neighborhood Interaction in a Homogeneous Community. American Sociological Review 15: 357–366.

[7] Butts CT, Acton RM, Hipp JR, Nagle NN (2011) Geographical Variability and Network Structure. Social Networks 34: 82–100.

[8] Durkheim E (1984 [1933]) The division of labour in society. HoundmillsBasingstokeHampshire: Macmillan. 352 p.

[9] Simmel G (1971) The Metropolis and Mental Life. In: Levine DN, editor. Georg Simmel: On Individuality and Social Forms. Chicago: University of Chicago.

[10] Wirth L (1938) Urbanism as a Way of Life. American Journal of Sociology 44: 1–24.

[11] Janowitz M (1952) The community press in an urban setting. GlencoeIL: Free Press. 256 p.

[12] Fischer CS (1975) Toward a Subcultural Theory of Urbanism. American Journal of Sociology 80: 1319–1341.

[13] Fischer CS (1982) To Dwell Among Friends: Personal Networks in Town and City. Chicago: University of Chicago.

[14] Sampson RJ (1991) Linking the Micro- and Macrolevel Dimensions of Community Social Organization. Social Forces 70: 43–64.

[15] Warner BD, Rountree PW (1997) Local Social Ties in a Community and Crime Model: Questioning the Systemic Nature of Informal Social Control. Social Problems 44: 520–536.

[16] Kasarda JD, Janowitz M (1974) Community Attachment in Mass Society. American Sociological Review 39: 328–339.

[17] Hipp JR, Perrin AJ (2006) Nested Loyalties: Local Networks' Effects on Neighborhood and Community Cohesion. Urban Studies 43: 2503–2523.

[18] Lowenkamp CT, Cullen FT, Pratt TC (2003) Replicating Sampson and Groves's Test of Social Disorganization Theory: Revisiting a Criminological Classic. Journal of Research in Crime and Delinquency 40: 351–373.

[19] Adams RE (1992) Is Happiness a Home in the Suburbs?: The Influence of Urban Versus Suburban Neighborhoods on Psychological Health. Journal of Community Psychology 20: 353–371.

[20] Campbell KE, Lee BA (1992) Sources of Personal Neighbor Networks: Social Integration, Need, or Time? Social Forces 70: 1077–1100.

[21] Logan JR, Spitze GD (1994) Family Neighbors. American Journal of Sociology 100: 453–476.

[22] Lee BA, Campbell KE, Miller O (1991) Racial Differences in Urban Neighboring. Sociological Forum 6: 525–550.

[23] Handy SL, Boarnet MG, Ewing R, Killingsworth RE (2002) How the built environment affects physical activity. American Journal of Preventive Medicine 23: 64–73. 12133739 10.1016/s0749-3797(02)00475-0

[24] Rodríguez DA, Khattak AJ, Evenson KR (2006) Can new urbanism encourage physical activity?: Comparing a new Urbanist neighborhood with conventional suburbs. Journal of the American Planning Association 72: 43–54.

[25] Talen E (1999) Sense of Community and Neighbourhood Form: An Assessment of the Social Doctrine of New Urbanism. Urban Studies 36: 1361–1379.

[26] Ahlbrandt RS (1984) Neighborhoods, People, and Community. New York: Plenum. 238 p.

[27] Lund H (2003) Testing the claims of new urbanism: Local access, pedestrian travel, and neighboring behaviors. Journal of the American Planning Association 69: 414–429.

[28] Richard L, Gauvin L, Gosselin C, Laforest S (2009) Staying connected: neighbourhood correlates of social participation among older adults living in an urban environment in Montreal, Quebec. Health Promot Int 24: 46–57. 19098293 10.1093/heapro/dan039 PMC5167566

[29] Rogers SH, Halstead JM, Gardner KH, Carlson CH (2010) Examining Walkability and Social Capital as Indicators of Quality of Life at the Municipal and Neighborhood Scales. Applied Research in Quality of Life 6: 201–213.

[30] Wood L, Shannon T, Bulsara M, Pikora T, McCormack G, et al (2008) The anatomy of the safe and social suburb: an exploratory study of the built environment, social capital and residents' perceptions of safety. Health Place 14: 15–31. 17576088 10.1016/j.healthplace.2007.04.004

[31] Hanibuchi T, Kondo K, Nakaya T, Shirai K, Hirai H, et al (2012) Does walkable mean sociable? Neighborhood determinants of social capital among older adults in Japan. Health Place 18: 229–239. 22000345 10.1016/j.healthplace.2011.09.015

[32] Volker B, Flap H, Lindenberg S (2007) When are neighborhoods communities? Community in Dutch neighborhoods. European Sociological Review 23: 99–114.

[33] James OW, Frederick PS (1971) Spatial dimensions of urban social travel. Annals of the Association of American Geographers 61: 371–386.

[34] Greenbaum SD, Greenbaum PE (1985) The Ecology of Social Networks in Four Urban Neighborhoods. Social Networks 7: 47–76.

[35] Groff E, McCord ES (2011) The Role of Neighborhood Parks as Crime Generators. Security Journal: 1–24.

[36] Knutsson J (1997) Restoring public order in a city park. In: Homel R, editor. Policing for Prevention: Reducing Crime, Public Intoxication and Injury Monsey, NY: Criminal Justice Press. pp. 133–151.

[37] Wickes R, Homel R, McBroom J, Sargeant E, Zahnow R (2011) Community Variations in Crime: A Spatial and Ecometric Analysis Wave 2: Technical Report No. 1 Study Methods and Basic Statistics. Brisbane: Australian Research Council.

[38] Mazerolle L, Wickes R, Cherney A, Murphy K, Sargeant E, et al.. (2012) Community Variations in Crime: A Spatial and Ecometric Analysis Wave 3: Technical Report No. 1 Study Methods and Basic Statistics. Brisbane: Australian Research Council.

[39] Mazerolle L, Wickes R, Rombouts S, McBroom J, T-K Shyy, et al.. (2007) Community Variations in Crime: A Spatial and Ecometric Analysis Wave 1: Technical Report No. 1 Study Methods and Basic Statistics. Brisbane: Australian Research Council.

[40] Raudenbush SW, Sampson RJ (1999) Ecometrics: Toward a science of assessing ecological settings, with application to the systematic social observation of neighborhoods. Sociological Methodology 1999, Vol 29. pp. 1–41.

[41] Sampson RJ, Morenoff JD, Earls F (1999) Beyond Social Capital: Spatial Dynamics of Collective Efficacy for Children. American Sociological Review 64: 633–660.

[42] Woldoff RA (2002) The Effects of Local Stressors on Neighborhood Attachment. Social Forces 81: 87–116.

[43] Sampson RJ (2013) The Place of Context: A theory and Strategy for Criminology's Hard Problems. Criminology 51: 1–31.

[44] Laurence J (2013) 'Hunkering Down or Hunkering Away?' The Effect of Community Ethnic Diversity on Residents' Social Networks. Journal of Elections, Public Opinion and Parties 23: 255–278.

[45] Wickes R, Zahnow R, White G, Mazerolle L (2013) Ethnic Diversity and its Impact on Community Social Cohesion and Neighbourly Exchange. Journal of Urban Affairs DOI: 10.1111/juaf.12015.

[46] Lancee B, Dronkers J (2011) Ethnic, religious and economic diversity in Dutch neighborhoods: Explaining quality of contact with neighbors, Trust in the neighborhood and inter-ethnic trust. Journal of Ethnic and Migration Studies 37: 597–618.

[47] Stolle D, Soroka S, Johnston R (2008) When Does Diversity Erode Trust? Neighborhood Diversity, Interpersonal Trust and the Mediating Effect of Social Interactions. Political Studies 56: 57–75.

[48] Leigh A (2006) Trust inequality and ethnic heterogeneity. The Economic Record 82: 268–280.

